# Magnetic Bead-Based Colorimetric Immunoassay for Aflatoxin B1 Using Gold Nanoparticles

**DOI:** 10.3390/s141121535

**Published:** 2014-11-14

**Authors:** Xu Wang, Reinhard Niessner, Dietmar Knopp

**Affiliations:** Institute of Hydrochemistry and Chemical Balneology, Chair for Analytical Chemistry, Technische Universität München, Marchioninistrasse 17, Munich 81377, Germany; E-Mails: xu.wang@tum.de (X.W.); reinhard.niessner@ch.tum.de (R.N.)

**Keywords:** aflatoxin B1, magnetic beads, gold nanoparticles, colorimetric detection, competitive immunoassay

## Abstract

A competitive colorimetric immunoassay for the detection of aflatoxin B1 (AFB) has been established using biofunctionalized magnetic beads (MBs) and gold nanoparticles (GNPs). Aflatoxin B1-bovine serum albumin conjugates (AFB-BSA) modified MBs were employed as capture probe, which could specifically bind with GNP-labeled *anti*-AFB antibodies through immunoreaction, while such specific binding was competitively inhibited by the addition of AFB. After magnetic separation, the supernatant solution containing unbound GNPs was directly tested by UV-Vis spectroscopy. The absorption intensity was directly proportional to the AFB concentration. The influence of GNP size, incubation time and pH was investigated in detail. After optimization, the developed method could detect AFB in a linear range from 20 to 800 ng/L, with the limit of detection at 12 ng/L. The recoveries for spiked maize samples ranged from 92.8% to 122.0%. The proposed immunoassay provides a promising approach for simple, rapid, specific and cost-effective detection of toxins in the field of food safety.

## 1. Introduction

Recently, nano-biotechnology, which integrates nanoscience with molecular biology and biomedicine, has attracted great attention in the field of biological analysis [1,2]. Nanoparticles of various shapes, sizes, and compositions, have been developed and extensively utilized, providing exciting possibilities for highly sensitive detection [3–7].

Magnetic beads (MBs) are attractive because they have good biocompatibility and can be easily separated from reaction mixture with the aid of an external magnet [8,9]. Iron oxide particles, for instance, have been widely used in immunoassay [10,11], protein and enzyme immobilization [12,13], cell separation [14], DNA hybridization [15,16] and drug delivery [17]. MB-based assays have been developed by combining MBs with recognition elements such as enzymes [18], noble metal nanoparticles [19,20], fluorescent nanoparticles [21,22] and carbon nanotubes [23]. Since the particles have a large surface area, diffuse freely in the reaction mixture and can be easily separated after reaction [24], these assays are usually fast and simple to operate, which offers a promising platform for the detection of various analytes such as proteins [25], DNA [26] and small molecules [27]. Different analytical methods have been employed in MB-based assays, including colorimetry [28], fluorescence [22,29], light scattering [30,31], electrochemistry [32], and chemiluminescence [10,33].

Among these methods, the colorimetric assay has gained considerable attention due to its incomparable advantages such as simplicity, practicality, rapidness and no requirement to utilize expensive or challenging instruments. Gold nanoparticles (GNPs) are extensively used in such colorimetric assays owing to their special physical and chemical properties, such as facile preparation, simplicity of modification and unique catalytic property [34]. For instance, GNPs serve as carrier to load active biomolecules like enzymes and artificial DNAzyme for enzyme-catalyzed signal amplification [35,36]. Further, GNPs are used as seeds for catalytic deposition of silver or gold, followed by scanometric or absorbance detection [37,38]. As another example, GNPs can catalyze the decomposition of organic dyes like methyl orange [39] and methylene blue [40] to generate colorimetric signals. Although high sensitivity might be achieved, the color development step in these assays indeed increased the complexity of assay. Obvious absorbance change might be also obtained even without amplification, because GNP itself has extremely high extinction coefficient. Liu *et al.* [41] developed a sandwich assay for DNA detection utilizing GNPs probes and MBs to recognize and capture the target DNA where the supernatant solution containing unbound GNPs was directly submitted to absorption measurements. A similar sandwich immunoassay was applied for protein detection [42], which proved a simple tool for bioanalysis.

Aflatoxins are highly toxic secondary metabolites produced mainly by *Aspergillus flavus* and *A. parasiticus*, present in a wide range of food products. More than 20 aflatoxins (e.g., B1, B2, G1, G2 and M1) have been identified. Among them aflatoxin B1 (AFB) is usually predominant in amount and constitutes the most hazardous, which is listed as Group I carcinogen by the International Agency for Research in Cancer [43]. Thus, various methods have been developed for the detection of AFB [44], like liquid chromatography coupled to mass spectrometry [45], high-performance liquid chromatography [46], immunochromatography [47] such as commercially available lateral flow strip assays [48,49], enzyme-linked immunosorbent assay (ELISA) [50] and electrochemical immunoanalysis [51]. Although these approaches are highly sensitive, more simpler and cost-effective methods are still desirable. Herein, we developed a competitive colorimetric immunoassay for AFB using MBs and GNPs. AFB-BSA-modified MBs (AFB-BSA-Fe_3_O_4_) and free AFB molecules competitively bind with GNP-labeled antibodies. After magnetic separation, the absorption of supernatant was measured directly, which is proportional to AFB concentration in sample. The influence of GNP size, pH and incubation time was tested. This method was applied for AFB determination in real maize samples.

## 2. Experimental Section

### 2.1. Materials and Reagents

Chloroauric acid (HAuCl_4_), sodium citrate, bovine serum albumin (BSA, fraction V, ∼99%), ferrous sulfate heptahydrate (FeSO_4_·7H_2_O), ammonium hydroxide (28% in water), polyoxyethylenesorbitan monolaurate (Tween-20), ochratoxin A (OTA), T-2 toxin, fumonisin B1 (FB1), AFB and AFB-BSA were purchased from Sigma Aldrich (Taufkirchen, Germany). Glutaraldehyde (25% in water) and potassium carbonate (K_2_CO_3_) were obtained from Merck (Darmstadt, Germany). Iron (III) chloride hexahydrate (FeCl_3_·6H_2_O), 3-aminopropyl-triethoxysilane (APTES, ∼99%), were purchased from Fluka (Buchs, Switzerland). Polyethylinglycol 8000 (PEG-8000) was purchased from Carl Roth (Karlsruhe, Germany). The mouse monoclonal antibody against AFB (*anti*-AFB, clone 1F2) was from our group [52]. Ultrapure water was produced using reverse osmosis with UV treatment (Milli-RO 5 Plus, Milli-Q185 Plus, Millipore, Eschborn, Germany). The absorption spectra were measured on UV/Vis spectrometer Specord 250 Plus (Analytik Jena, Jena, Germany). Dynamic light scattering (DLS) measurements were performed on a NANO-flex particle size analyzer (Microtrac, Meerbusch, Germany).

### 2.2. Preparation of AFB-BSA-Modified Magnetic Beads (AFB-BSA-Fe_3_O_4_)

Fe_3_O_4_ MBs were synthesized according to a previously reported method [23]. In brief, 1.35 g of FeCl_3_·6H_2_O and 0.695 g of FeSO_4_·7H_2_O were dissolved in 25 mL of water. The solution was heated at 80 °C for 10 min. Then 2 mL of ammonia (28% in water) was added. After stirring at 80 °C for 30 min, the obtained black particles were separated using an external magnet and washed thoroughly with water and ethanol. The particles were dried at 120 °C for 30 min. Then 60 mg of Fe_3_O_4_ particles were dispersed in 10 mL of ethanol under sonication for 15 min. 100 μL water and 200 μL APTES were added. The suspension was shaken for 5 h at room temperature (RT). The APTES-modified Fe_3_O_4_ particles (NH_2_-Fe_3_O_4_) were separated and washed thoroughly to get rid of any physically adsorbed APTES. The amino-functionalized particles were dispersed in 4 mL water.

One milliliter of NH_2_-Fe_3_O_4_ (∼15 mg/mL) was washed and dispersed in 1 mL of phosphate buffer solution (PBS, pH 8.0, 50 mM, prepared by using 0.05 M NaH_2_PO_4_ and 0.05 M Na_2_HPO_4_). Then 0.5 mL of glutaraldehyde (25% in water) was added and the suspension was shaken for 1 h at 100 rpm. The aldehyde-activated particles were separated, washed and redispersed in 1 mL 50 mM PBS (pH = 8). Finally 50 μL of AFB-BSA (1.5 mg/mL in water) was added and the suspension was well mixed and then shaken overnight. To block free sites on MBs' surface, 100 μL of BSA solution (50 mg/mL) was added and the mixture was shaken for another 1 h. The obtained AFB-BSA-Fe_3_O_4_ particles were washed five times with water, dispersed in 5 mL water and stored at 4 °C when not in use. The concentration of AFB-BSA-Fe_3_O_4_ was ∼3 mg/mL. Scheme 1a schematically illustrates the conjugation process.

### 2.3. Preparation and Functionalization of GNPs (Ab-GNPs)

To avoid any unwanted nucleation and aggregation during the synthesis, all glassware and stirrer were cleaned thoroughly with aqua regia (HNO_3_-HCl, 1:3, v/v), and then washed with water before use. GNPs with different size were prepared according to the Frens method [53]. Briefly, a solution of sodium citrate was quickly added to a boiled HAuCl_4_ solution (0.01%, 50 mL) under vigorous stirring. The color changed from yellow over black to red and the solution was kept boiling and stirred for further 15 min. Then the colloid solution was cooled to RT and filtered through a syringe filter with pore size of 220 nm. The obtained GNPs were stored at 4 °C in the refrigerator. Different size GNPs were prepared by changing the amount of sodium citrate. The GNPs were characterized by UV-Vis spectroscopy and DLS measurements.

Antibody-conjugated GNPs (Ab-GNPs) were prepared following a previously reported procedure with a few modifications [54]. Scheme 1b represents the fabrication of Ab-GNPs. First, the pH value of GNPs solution was adjusted to 8∼9 by adding 0.1 M K_2_CO_3_. 50 μL of *anti*-AFB antibody (1 mg/mL in 10 mM PBS containing 0.8% NaCl) was added to 5 mL of pH-adjusted GNPs solution. After incubation for 1 h at RT, 570 μL of 5% BSA was added to block any free binding sites on GNPs' surface. 1 h later, Tween-20 was added to a final concentration of 0.1% (w/v) for stabilizing the GNPs. The excess antibody, BSA and Tween-20 were removed by centrifugation at 9500 *g* for 15 min at 4 °C. The pink supernatant was carefully removed. The oily ruby sediment was washed twice and finally dispersed in 4 mL washing solution (5 mM PBS, pH 7.4, 0.1% PEG-8000). The antibody modified GNPs were stored at 4 °C.

### 2.4. Optimization and Detection Procedures

General procedure for the detection of AFB: 100 μL of AFB standard solution in 50 mM PBS at varied concentrations were mixed with 100 μL of Ab-GNPs, and then 20 μL of AFB-BSA-Fe_3_O_4_ suspension (∼3 mg/mL) were added. The mixture was well mixed and incubated at RT for 30 min with a rotation speed of 200 rpm on a shaker. After magnetic separation of the formed immune-complexes (*i.e.*, GNP-*anti*-AFB-AFB-BSA-Fe_3_O_4_), the supernatant was directly transferred into a cuvette for UV-Vis measurements. The absorbance maximum was recorded and final absorbance was calculated by subtracting the absorbance of the corresponding blank samples. Each immunoassay was conducted three times to determine the reproducibility. The principle of the competitive immunoassay is illustrated in Scheme 1c.

To study the influence of GNP size on the performance of AFB detection, antibody-modified GNPs with three different sizes were used. To investigate the effect of pH value on testing results, AFB standard solutions were prepared in three different pH buffers: 6.0, 7.4 and 8.2 at 50 mM. To test the influence of incubation time on the immunoassay, mixed solution with 20 μL AFB-Fe_3_O_4_, 100 μL Ab-GNPs (34.8 nm) and 100 μL PBS (50 mM, pH 7.4) were incubated for 2 to 60 min and separated at corresponding time.

To evaluate the selectivity of the developed approach, OTA, T-2 toxin, FB1 and their mixtures with AFB were tested. The concentration of all other mycotoxins used was 20 ng/mL, while the concentration of AFB was 1 ng/mL.

### 2.5. Preparation of Maize Samples

Maize samples were purchased from a local market. 1 g of the pulverized maize samples was spiked with AFB (in methanol) at concentrations of 0, 5, 10, 20 and 50 μg/kg, respectively. The spiked samples were kept overnight at RT in a light-protected fume hood to evaporate methanol. Then the spiked samples were extracted with 5 mL of methanol–water (80:20, v/v) by vortex mixing for 3min and then centrifugated at 1920 *g* for 15 min [47]. The supernatant was 20-fold diluted with PBS (50 mM, pH = 7.4) for colorimetric assays.

## 3. Results and Discussion

### 3.1. Characteristics of MBs and GNPs

To construct a double-bead competitive immunoassay, antibody and antigen are normally immobilized on different beads, respectively. If MBs are used as carrier for antibody, usually the surface of MBs is first modified by linker proteins such as protein A, G and streptavidin [18,31], to avoid activity loss of antibody after immobilization. However, the activity of an antibody will be only minimal affected when it is labeled directly with colloidal gold [10]. In this assay, the stability of AFB-BSA on MBs is much higher compared to the antibody (data not shown). Therefore, also in consideration of the cost and complexity, we immobilized antibodies on GNPs while AFB-BSA molecules were covalently linked to MBs.

**Figure 1.**
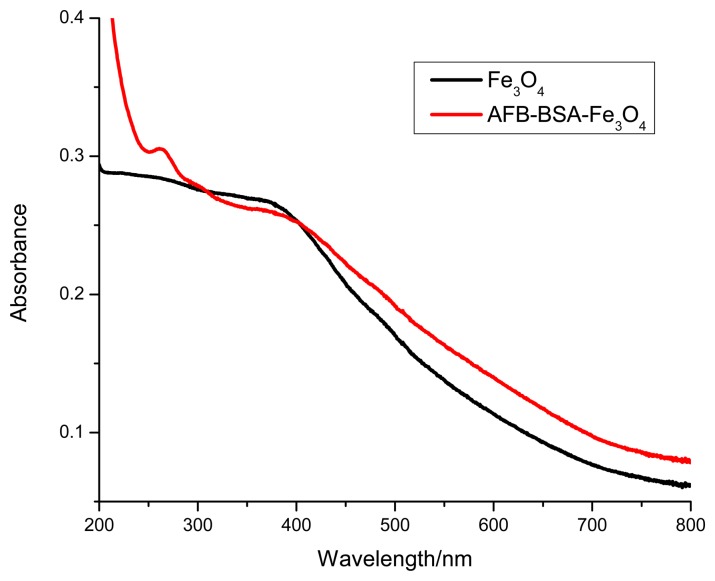
UV-Vis absorption spectra of Fe_3_O_4_ and AFB-BSA-Fe_3_O_4_ particles.

The Fe_3_O_4_ MBs were prepared by co-precipitating trivalent and divalent iron ions in alkaline solution under heating. Then primary amino groups were introduced by the reaction with APTES. Finally AFB-BSA conjugates were covalently immobilized on the surface of MBs through the cross-linking of glutaraldehyde. The MBs not only serve as solid carrier, yet facilitate the rapid separation of immune-complexes. As shown in Figure 1, the absorbance of bare Fe_3_O_4_ particles steadily decreased from 200 to 800 nm. After reaction with AFB-BSA conjugate, an obvious absorption peak appeared between 250 and 300 nm, which indicates the successful immobilization of protein on the surface of magnetic particles. The average size of AFB-BSA-Fe_3_O_4_ was ∼3.3 μm as determined by DLS. The larger size might be ascribed to agglomeration of small particles under heating.

GNPs of different sizes were prepared through the reduction of HAuCl_4_ by sodium citrate. The particle size was determined by DLS. As shown in Table 1, the average size of GNPs could be well tuned by the amount of reduction reagent. As the volume of sodium citrate decreasing from 1 to 0.5 mL, the particle size grows from 25.3 to 49.0 nm, and the surface plasmon resonance peak, λ (SPR), red shifts from 524 to 538 nm, which is consistent with the reported results [55].

After being coated with antibodies, the hydrodynamic diameter of GNPs increases correspondingly. The thickness increase, of ∼10 nm, is almost equal to the size of antibody, which indicates successful attachment of antibodies on the colloidal surface. The slight bathochromic shift in plasmon band position also confirms the formation of bioconjugates. The association of antibody to the colloidal surface was possibly due to direct coupling of functional groups of protein with gold through electrostatic interactions and coordination effect, such as cysteine, NH_3_^+^-lysine residues and imidazole groups on antibody [28].

### 3.2. Influence of GNP Size on the Immunoassay

**Figure 2.**
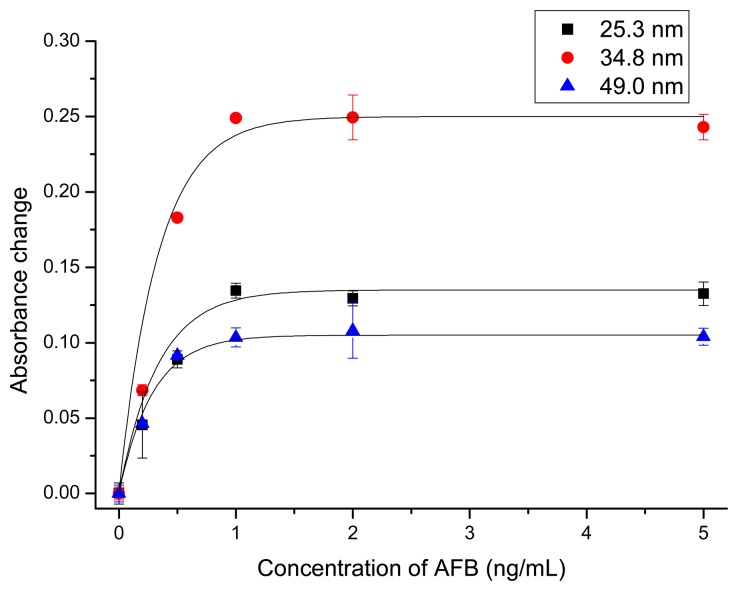
Dose-response curves for the detection of AFB using different size of GNPs.

Since the SPR characteristic of GNPs is dependent on the GNP radius, the effect of the GNP size on the assay sensitivity was investigated. There are two opposite trends, which favor large and small GNPs, respectively. That is, with the increase of particle size, the extinction coefficient of GNP increases exponentially. The removal of a single, large GNP will lead to a larger absorbance change compared to the removal of smaller ones [42]. On the other hand, due to the larger surface-to-volume ratio of smaller GNPs, they are more effectively functionalized with antibodies. Further, because of the smaller steric hindrance when binding with magnetic beads, smaller GNPs might give better response. Based on this consideration, we tested GNPs of different sizes for the detection of AFB. The corresponding dose–response curves are shown in Figure 2. The three kinds of GNPs gave similar response. With the increase of AFB concentration, the absorbance of supernatant increased correspondingly. The saturation level was obtained at a concentration of ∼1 ng/mL AFB. At this concentration the absorbance change at SPR was 0.11, 0.25 and 0.13 for GNPs of 25.3, 34.8 and 49.0 nm, respectively. Since 34.8 nm GNPs gave the largest signal change, they were used in further experiments.

### 3.3. Optimization of Experimental Conditions

**Figure 3.**
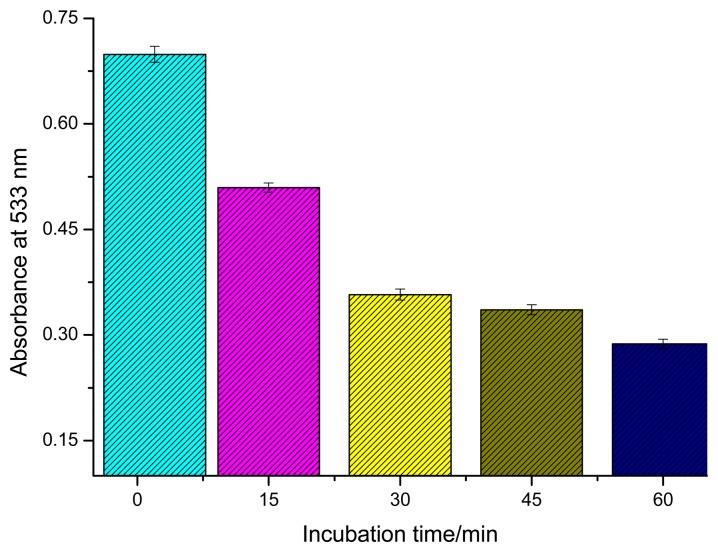
The effect of incubation time on the absorbance of supernatant.

The performance of the developed immunoassay could be greatly affected by parameters such as temperature, incubation time and pH. To simplify the analytical procedure, all experiments were conducted at RT. The effect of various incubation times was studied. Figure 3 shows the absorbance changes of supernatant after different incubation times when Ab-GNPs were incubated with AFB-BSA-Fe_3_O_4_ in PBS (pH = 7.4, 50mM). The absorbance decreased with increasing incubation time, which indicates that functionalized GNPs could gradually bind with the magnetic beads *via* antigen-antibody reaction, leading to a lower concentration of GNPs in bulk solution after magnetic separation. During the first 30 min, the absorbance decreased rapidly while it changed less between 30 and 60 min. Due to limited diffusion of biofunctionalized nanoparticles, the final equilibrium of the immunoreaction might not be reached within 30 min. Nevertheless, a 30 min incubation was chosen in this study because there was only a small difference of absorbance between 30 and 60 min.

**Figure 4.**
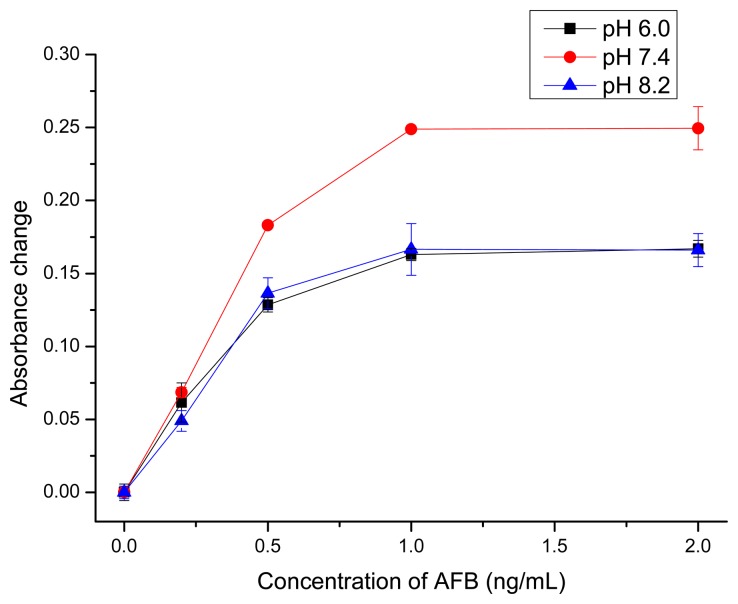
Optimization of pH value for the detection of AFB.

The net charges of biomolecules (antibody and AFB-BSA) are affected by the pH value. The possible interactions between AFB and antibody such as hydrogen bond and hydrophobic interaction [56] might also change at different pH. So the influence of pH was investigated. As the isoelectric point of AFB antibodies is ∼pH 7.0 and the isoelectric point of BSA is ∼pH 4.7, the antibodies are positively charged, while BSA molecules are negatively charged at pH 6.0. This could lead to nonspecific adsorption of Ab-GNPs to AFB-BSA-Fe_3_O_4_ particles' surface via electrostatic attractions, which causes a relatively low sensitivity, as shown in Figure 4. When the pH was too high (pH = 8.2), there might be electrostatic repulsions between Ab-GNPs and AFB-BSA-Fe_3_O_4_ particles since both antibody and BSA were negatively charged, which also gave a lower sensitivity. The highest sensitivity was obtained at pH 7.4, when antibodies carried almost no net charges. Thus, PBS buffer with pH 7.4 was used for following quantitative analysis.

### 3.4. Measurement of AFB

**Figure 5.**
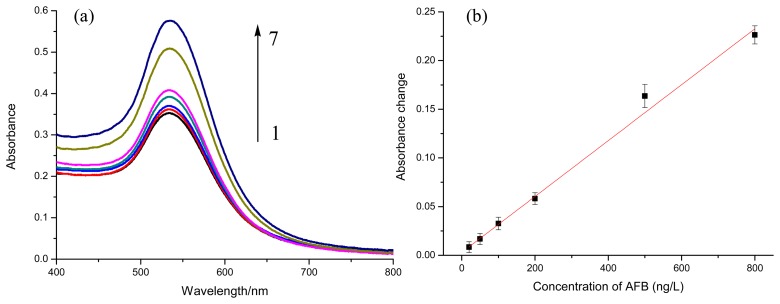
(**a**) UV-Vis absorption spectra of supernatant after magnetic separation with different concentrations of AFB (from sample 1 to 7: 0, 20, 50, 100, 200, 500, 800 ng/L) (**b**) the relationship between absorbance changes and AFB concentration (*n* = 5).

AFB in PBS at different concentrations (0, 20, 50, 100, 200, 500, 800 ng/L) were quantitatively analyzed under optimal conditions, *i.e.*, 20 μL of AFB-BSA-Fe_3_O_4_ (3 mg/mL), 100 μL of Ab-GNPs (34.8 nm) at pH 7.4 with incubation time of 30 min. Figure 5a shows the absorption spectra of supernatant after magnetic separation. With the increase of AFB concentration, the absorbance at 533 nm increased correspondingly. The absorbance change at 533 nm was linear to AFB concentration in the range of 20 to 800 ng/L (ΔAbs = 2.87 × 10^−4^C_AFB_ + 0.00298) with a correlation coefficient of 0.992 (Figure 5b). The limit of detection was calculated to be 12 ng/L using three times of signal-to-noise ratio. The reproducibility of the immunoassay was evaluated by calculating the intra- and inter-batch variation coefficients (CVs, *n* = 3). Experimental results indicated that the CVs of the assays using biofunctionalized nanoparticles from the same batch were 5.0%, 1.6%, and 1.7% at 0.1, 0.2, and 0.8 ng/mL AFB levels, respectively, while the CVs of the assays using particles from different batches were 11.6%, 8.7%, and 3.0% at the above-mentioned analyte concentrations.

The detectable concentration range is about one order of magnitude lower than that of MB-based fluorescence immunoassay (0.5–30 ng/mL) [21] and homogeneous immunoassay based on competitive dispersion of gold nanorods (0.5–20 ng/mL) using the same antibody [57]. Although the developed method is not as sensitive compared to some other ELISAs or electrochemical immunosensors, its simplicity and applicability are indeed attractive.

**Figure 6.**
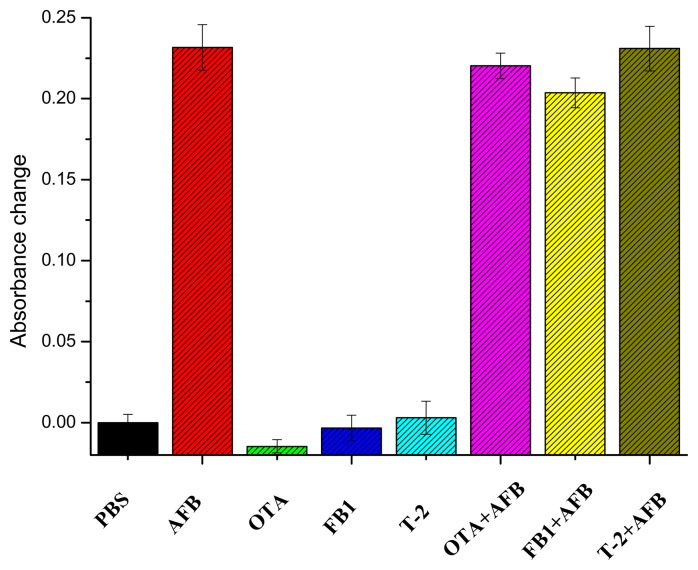
The selectivity of AFB determination using the developed immunoassay.

The selectivity and specificity of the established approach was also investigated. OTA, FB1, T-2, and their mixture with AFB were analyzed by the developed immunoassay. As indicated in Figure 6, the presence of other mycotoxins caused very small signal change, while the signal change was almost the same as AFB alone compared with that of mixtures containing AFB and the interfering agents, indicating a negligible impact of the other tested mycotoxins.

### 3.5. Analysis of AFB Spiked Maize Samples

To further evaluate analytical reliability and possible application of the established assay, maize samples artificially spiked with AFB were analyzed. Non-spiked sample extract dilution was used as blank and AFB concentration in maize samples were determined from the calibration curve. The testing results are listed in Table 2. Acceptable recovery was obtained in the range of 92.8% to122.0%. Thus, the established immunoassay could be useful for the determination of AFB in real samples.

## 4. Conclusions

In summary, we have demonstrated the feasibility of a competitive colorimetric immunoassay for the determination of AFB using AFB-BSA-modified magnetic particles as capture probe and *anti*-AFB coated GNPs as signal probe. The whole assay is simple, time-saving and cost-effective, owing to rapid separation by the use of magnetic particles and no requirement of color development step. The proposed approach allows the detection of AFB within the low ng/L range in PBS and real maize sample extract. This method could be easily extended for the detection of other mycotoxins since the biofunctionalization process is very simple and generally applicable. Multiple-analyte detection could be established by simultaneously using different metal nanoparticles such as gold nanorods and silver nanoparticles.

## Figures and Tables

**Figure:**
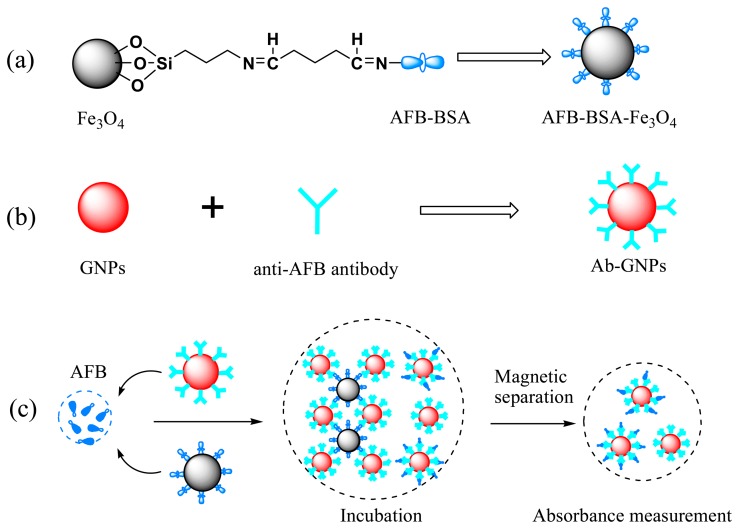
**Scheme 1.** Schematic illustration of the preparation of (**a**) Aflatoxin B1-bovine serum albumin (AFB-BSA)-Fe_3_O_4_; (**b**) antibody coated gold nanoparticles (GNPs); and (**c**) principle of the competitive colorimetric immunoassay for AFB detection.

**Table 1.** The preparation and physical properties of GNPs.

**Sample**	**HAuCl_4_ (0.01%)**	**Sodium Citrate (1%)**	**Citrate-Stabilized**		**Antibody-Coated**	
	
			**DLS Size**	**λ (SPR)**	**DLS Size**	**λ (SPR)**
GNP 1	50 mL	1 mL	25.3 nm	524 nm	44.0 nm	529 nm
GNP 2	50 mL	0.75 mL	34.8 nm	529 nm	55.4 nm	533 nm
GNP 3	50 mL	0.5 mL	49.0 nm	538 nm	74.2 nm	543 nm

**Table 2.** The detected results of AFB in maiz samples and recoveries (*n* = 3).

**In Spiked Sample (μg/kg)**	**After Dilution (ng/L)**	**Detected Concentration (ng/L)**	**RSD (ng/L)**	**Recovery(%)**
5	50	57.7	11.5	115.4
10	100	122.0	16.4	122.0
20	200	192.1	24.2	96.1
50	500	464.2	40.4	92.8
